# Regenerative Organic Agriculture and Human Health: The Interconnection Between Soil, Food Quality, and Nutrition

**DOI:** 10.3390/antiox14050530

**Published:** 2025-04-29

**Authors:** Giulia Feliziani, Laura Bordoni, Rosita Gabbianelli

**Affiliations:** 1 School of Advanced Studies, University of Camerino, 62032 Camerino, Italy; giulia.feliziani@unicam.it; 2 Unit of Molecular Biology and Nutrigenomics, University of Camerino, 62032 Camerino, Italy; laura.bordoni@unicam.it

**Keywords:** soil health, antioxidants, polyphenols, food nutrient density, bioactive compounds, sustainable agriculture, chronic disease prevention

## Abstract

Regenerative organic agriculture (ROA) combines ecological and organic principles to promote soil health, biodiversity, and long-term sustainability. This narrative review explores the connection between soil quality, food nutritional value, and human health, highlighting how ROA can enhance phytochemical content and reduce harmful residues in plant-based foods. Empirical studies report increases in vitamin C, zinc, and polyphenols in crops such as leafy greens, grapes, and carrots grown under regenerative systems, along with reductions in nitrates and pesticide residues. We summarize recent literature (2000–2025) that links soil-driven improvements in food composition to antioxidant activity and potential health benefits. By addressing current research gaps, this review supports the role of ROA in building resilient food systems and preventing chronic disease.

## 1. Introduction

Nutrition plays an essential role in chronic disease prevention, immune system support, and general well-being [1,2]. A diet rich in nutrients, antioxidants, and bioactive compounds can reduce the risk of cardiovascular disease, diabetes, and cancer [3]. Among these compounds, polyphenols and flavonoids help fight inflammation and oxidative stress. Since the nutritional quality of food depends on soil health and agricultural practices, improving soil with sustainable methods is critical to overall health [4]. Regenerative agriculture (RA), a holistic system that seeks to restore soil and ecosystem health, has gained increasing attention for its potential to improve food quality and sustainability [5].

Over past decades, intensive agricultural practices have drastically altered the global landscape, leading to widespread soil degradation and presenting significant challenges to agricultural productivity, biodiversity, and environmental sustainability [6,7]. The excessive use of chemical fertilizers and pesticides, along with monoculture farming, has not only reduced soil fertility but also disrupted the microbial communities essential for soil health [8]. While these practices initially boosted yields, they initiated a cycle of diminishing returns, greater dependency on inputs, and escalating environmental harm. Simultaneously, global population growth and shifting dietary demands have placed unprecedented pressure on agricultural systems, further amplifying the need for productivity [9].

The intricate relationship between agricultural practices, soil health, food quality, and human health has emerged as a key focus in scientific research. Soil quality, characterized by its chemical composition, structure, and microbial biodiversity, is fundamental to determining the nutritional, chemical, and sensory properties of crops [10]. Healthy soils support robust plant growth and foster the production of bioactive compounds, which are essential to the functional and nutritional attributes of food [11]. Conversely, degraded soils result in nutrient-poor crops, reduced yields, and increased susceptibility to pests and diseases, thereby exacerbating the challenges faced by conventional farming systems [10].

The global trajectory of agricultural expansion further heightens these challenges, posing serious long-term risks to ecosystems through deforestation, biodiversity loss, and greenhouse gas emissions. These interconnected issues cast doubt on the capacity of conventional agricultural systems to secure long-term food security and nutrition [12].

In the face of these pressing challenges, sustainable agricultural practices have gained prominence as both a necessity and an opportunity. Consumers increasingly demand foods that are not only nutritionally superior but also sustainably produced, reflecting evolving societal values centered on health and environmental responsibility [13,14,15]. This dual emphasis on sustainability and health underscores the need for a deeper understanding of the dynamic relationships between soil health, agricultural practices, and food quality.

Sustainable farming approaches, such as organic and regenerative agriculture, have emerged as promising solutions to these challenges. By prioritizing soil health through reduced chemical inputs, enhanced microbial biodiversity, and practices like crop rotation, cover cropping, and minimal tillage, these methods restore soil fertility while producing safer, nutrient-rich foods. Importantly, these approaches align with consumer preferences for high-quality, environmentally sustainable products, offering viable pathways to address the interconnected crises of nutrition, environmental degradation, and food security [14,15].

This narrative review examines the critical role of organic and regenerative agricultural practices in addressing these global challenges. By synthesizing current scientific evidence published between 2000 and 2025, it explores how these approaches contribute to improving soil health, protecting the environment, and enhancing the nutritional and functional qualities of food. Although previous reviews have addressed sustainable agriculture or the nutritional quality of organic foods, few have integrated the direct link between regenerative organic agriculture (ROA), soil-driven phytochemical enhancement, and health implications. This review aims to fill that gap by connecting ROA practices with antioxidant pathways and chronic disease prevention, providing novel insights into how soil-focused interventions may benefit human health and sustainability.

## 2. Regenerative Agriculture

### 2.1. What Is Regenerative Agriculture

The concept of regenerative agriculture (RA) was introduced in the 1980s by Robert Rodale [16]. In recent years, it has gained increasing attention and popularity [17,18]. However, defining it precisely is not straightforward, as its application varies according to geographical and environmental context [18,19,20,21].

RA is a multidisciplinary concept addressing the environmental, economic, and social dimensions of farming, reflecting its broad scope and adaptability. Despite extensive research efforts, defining RA in a universally accepted manner remains challenging due to its complexity and context-specific nature. A recent meta-analysis by Jayasinghe and colleagues provides a comprehensive definition, describing RA as a transdisciplinary approach that integrates local and indigenous knowledge with established scientific insights [22]. This framework combines adaptable principles with context-specific practices, prioritizing soil conservation as a foundational step to restore soil health, enhance ecosystem functions, and improve socioeconomic outcomes.

Unlike organic farming, which strictly prohibits synthetic inputs, RA adopts a more flexible approach, promoting their minimal and responsible use when necessary [20]. This strategy allows farmers to focus on restoring soil health and biodiversity while gradually reducing dependence on synthetic fertilizers and pesticides [23]. Its central aim is to restore and strengthen the health, vitality, and adaptive capacity of interconnected ecosystems within farms, ensuring their long-term sustainability [23]. Furthermore, it emphasizes the social and economic dimensions of food production systems, aiming to foster long-term resilience in social, economic, and environmental contexts [18,24]. Case studies in North America and Europe have shown that farms adopting RA practices report improved crop yields, enhanced soil fertility, and increased financial viability due to their reduced dependence on chemical inputs [25,26].

A significant focus of RA is on improving soil health, particularly by restoring organic carbon levels. As Sahu and Das explain, regenerative practices leverage carbon absorbed by plants from the atmosphere to rebuild soil organic carbon, thereby promoting resilience [27]. In addition to soil restoration, RA seeks to strengthen economic stability within farming communities, enhance water management, and conserve biodiversity, both above and below ground. This holistic approach underscores RA’s potential to address interconnected global challenges while adapting to the unique characteristics of diverse landscapes and farming systems.

Driven by the projected global population of 8.6 billion by 2030, intensified conventional industrial agriculture practices exacerbate resource exploitation and environmental degradation, posing significant sustainability challenges in meeting the surging food demand [22]. This concern has fueled interest in developing more sustainable farming systems that reduce synthetic input dependency. RA offers the synergistic potential of landscape restoration and biodiversity conservation [22]. RA practices provide a promising avenue to reverse the trend of degrading landscapes, holding substantial potential for the future of global sustainable agriculture [22].

This approach has gained growing attention in response to global challenges related to climate change and food security. One of its primary benefits is its capacity to sequester organic carbon in the soil, contributing to greenhouse gas emission mitigation [28]. Soil can function as both a source and a sink of carbon, depending on management, biomass input levels, micro-climatic conditions, and bioclimatic change. Global soils are estimated to store approximately 1500 gigatons of carbon, three times the amount present in the atmosphere, making improved soil management a crucial factor in combating global warming [28,29].

RA enhances the resilience of agricultural systems against extreme weather events, such as droughts and floods. Practices such as reduced tillage and cover cropping help preserve soil structure, improve moisture-holding capacity, and protect crops from the adverse effects of climatic fluctuations. Studies from drought-prone regions have demonstrated that farms implementing regenerative practices experience higher soil moisture retention and improved crop survival rates during extreme weather events [30,31]. By integrating these strategies, RA stands as a viable solution for modern agricultural and environmental challenges, offering a sustainable path forward for food production and ecosystem conservation.

RA is not only a response to environmental challenges but also an opportunity to transform agricultural systems into a more resilient and productive model. However, its implementation requires initial investment, technical training, and institutional support to incentivize farmers to adopt these practices. Only through an integrated approach, involving research, public policy, and local communities, will it be possible to maximize the benefits of regenerative agriculture on a global scale [32].

### 2.2. Key Principles and Practices

**Figure 1:**
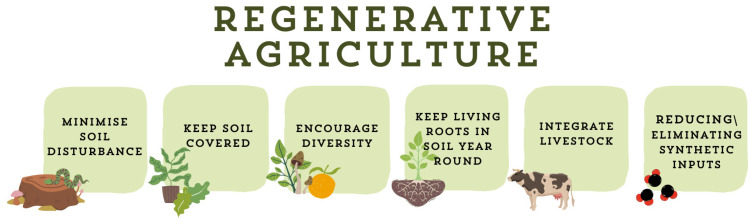
The fundamental principles of RA: maintaining continuous soil cover, minimizing soil disturbance, preserving living roots in the soil throughout the year, increasing species diversity, integrating livestock, and reducing or eliminating synthetic inputs like herbicides and fertilizers. Original figure created by the authors.

Figure 1 shows the fundamental principles of RA. RA focuses on creating sustainable and resilient agricultural systems by maintaining continuous soil cover, minimizing soil disturbance, preserving living roots in the soil throughout the year, increasing species diversity, integrating livestock, and reducing or eliminating synthetic inputs like herbicides and fertilizers [23,32]. These practices, some of which have been used by farmers for generations, are deeply rooted in ecological science, while others are more recent innovations requiring technological support and investments in specialized machinery.

A key aspect of RA is reducing soil disturbance. Practices such as reduced tillage or zero tillage are essential for preserving soil structure, which plays a critical role in nutrient cycling. By allowing fungal hyphae to proliferate and maintaining the integrity of soil aggregates, these techniques help reduce erosion and retain soil organic matter [18]. An intact soil structure also significantly improves water retention, enabling the soil to store more water for plant use [33]. This is especially important in mitigating the effects of drought, making agricultural systems more resilient to climate variability.

Maintaining vegetation cover is another crucial practice. Studies have demonstrated that during periods when the main crop is not growing, planting cover crops, such as legumes (e.g., clovers, lentils, beans) and grasses (e.g., ryegrass, barley, oats), plays a crucial role in soil conservation by enhancing soil structure, improving fertility, reducing soil erosion, water evaporation, and promoting better water retention [34,35,36]. For instance, a field trial in Canada showed that using winter rye as a cover crop reduced soil erosion by 40% and improved water retention by 25% [35]. Additionally, cover crops regulate soil temperature, creating a favorable environment for microbial life [23]. These microbial communities play a pivotal role in nutrient cycling and overall soil health [35].

Promoting biodiversity is fundamental to regenerative systems. Crop diversification, intercropping, and integrating natural habitats within farmland enhance ecological resilience and disrupt cycles of pests and diseases [34,37,38,39]. Rotating diverse crops not only minimizes soil degradation associated with monoculture but also improves nutrient cycling and supports the development of a healthy, balanced soil ecosystem [40,41,42]. Integrating livestock into crop systems through practices like rotational grazing further contributes to nutrient recycling and soil fertility, mimicking natural processes that sustain ecosystems [23]. A recent review by Lai and colleagues further highlights the benefits of intercropping as a strategy to optimize resource use and improve soil health [43]. By allowing crops to efficiently share sunlight, water, and nutrients, intercropping enhances yield stability and land productivity while reducing weed competition, pest outbreaks, and soil depletion [43]. Legume–cereal intercropping systems are particularly valuable, as legumes fix atmospheric nitrogen and transfer it to cereals, improving soil fertility. Additionally, intercropping fosters a more diverse soil microbial community, influencing nitrogen cycling and plant–microbe interactions [43].

Maximizing nutrient and water-use efficiency is another priority. Reducing the reliance on chemical fertilizers and pesticides in favor of organic inputs like compost and manure preserves water quality and enhances soil structure [44,45]. Techniques such as contour plowing and planting cover crops help reduce water runoff and soil erosion, while grasses planted along waterways stabilize the soil, preventing sedimentation and protecting aquatic ecosystems [46].

Livestock integration, particularly through rotational grazing, exemplifies the holistic management approach central to RA. By rotating animals through a series of paddocks, overgrazing is avoided, manure is evenly distributed as a natural fertilizer, and soil health is improved [47]. This approach not only increases soil organic carbon but also supports ecosystem services such as carbon sequestration [47]. While livestock farming is often associated with methane emissions, properly managed grazing systems provide significant environmental benefits, including enhanced soil fertility and resilience [5,23]. For example, in a study conducted in the Midwest USA, farms adopting RA practices, such as minimal tillage and cover cropping, demonstrated a 20% increase in soil organic matter and a 30% reduction in fertilizer use over five years [20]. Similarly, in France, vineyards implementing regenerative methods reported improved grape quality and higher resilience to drought conditions [21].

Ultimately, RA leverages a combination of traditional wisdom and modern innovations to restore soil health, improve water efficiency, and promote ecological balance. By focusing on these practices, farmers can build more sustainable and resilient systems that benefit both agricultural productivity and the environment.

Another emerging practice in regenerative systems is the application of biochar, a stable carbon-rich material obtained from the pyrolysis of plant biomass. When incorporated into soil, biochar can enhance soil structure, water retention, and nutrient availability, while also supporting microbial biodiversity [23]. Recent studies suggest that its use may positively affect crop nutritional quality by increasing the concentration of certain micronutrients and phytochemicals [48].

**Figure 2:**
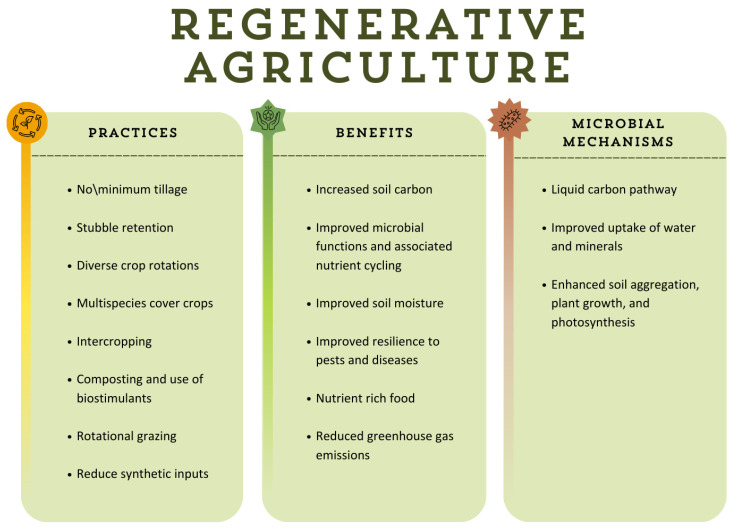
Fundamental practices of RA, benefits, and mechanisms that contribute to improved nutrient cycling, plant growth, and ecosystem resilience [23]. Original figure created by the authors.

Figure 2 provides an overview of the fundamental principles and practices of RA, highlighting their benefits for soil health, biodiversity, and sustainability. It also illustrates the key microbial mechanisms that contribute to improved nutrient cycling, plant growth, and ecosystem resilience.

### 2.3. Regenerative Organic Agriculture

The integration of regenerative and organic agriculture (ROA) offers a synergistic approach that significantly enhances soil health, food quality, environmental sustainability, and socio-economic outcomes [49,50,51,52]. Organic farming prioritizes the avoidance of synthetic inputs, such as fertilizers and pesticides, thereby preserving soil microbial biodiversity, while RA focuses on soil restoration through practices like cover cropping, crop rotation, and reduced tillage [53]. Together, these methods build soil organic matter, improve nutrient cycling, enhance water retention, and promote long-term soil fertility [50,53]. Crops cultivated under organic systems are often richer in nutrients and bioactive compounds, as the absence of synthetic chemicals facilitates the uptake of nutrients from healthier soils [8,54]. Regenerative methods further contribute to improved nutritional profiles and sensory qualities of food by fostering soil vitality [50,55].

The combination of these approaches also bolsters biodiversity by eliminating harmful synthetic inputs and integrating agroecological practices that support diverse ecosystems both above and below ground [49]. This enhanced biodiversity creates a resilient agricultural system, benefiting pollinators, natural pest control, and soil microbiomes. Additionally, these systems play a vital role in climate change mitigation; organic farming reduces greenhouse gas emissions by avoiding energy-intensive synthetic fertilizers, while regenerative practices, such as agroforestry and cover cropping, sequester carbon in the soil [32,56]. This combined strategy reduces the carbon footprint of agriculture and enhances climate resilience [28,50,57].

Economically, the rising demand for organic and sustainably produced foods provides premium market opportunities, while the reduced reliance on costly external inputs in regenerative practices improves farm profitability [58,59]. Both systems emphasize the importance of local and traditional knowledge, empowering farmers and fostering community engagement. Furthermore, these practices significantly reduce environmental impacts by minimizing soil and water contamination from agrochemicals, restoring degraded lands, and preventing soil erosion, thereby protecting ecosystems and ensuring sustainable agricultural productivity [55].

Organic and regenerative methods also increase resilience to environmental stressors by promoting robust soil and plant health, enabling crops to better withstand pests, diseases, and climatic variability [49,60]. This ensures sustainable yields, even in challenging conditions. Finally, the principles of ROA align closely with growing consumer demand for sustainable, high-quality products and with policy initiatives that incentivize environmentally friendly practices, providing additional support for their adoption and contributing to a more sustainable agricultural future [50,59].

By integrating ROA principles, agricultural systems can achieve a balance between productivity, sustainability, and environmental stewardship, addressing both current and future challenges in global food production.

## 3. Links Between Soil Health and Food Quality

### 3.1. Links Between Soil Health and Plant Health

The intricate relationship between soil composition and plant development is a dynamic interplay of physical, chemical, and biological factors. Soil health refers to the condition of the soil in terms of its capacity to sustain biological productivity, enhance environmental quality, and support both plant and animal health [11]. Soil properties, including texture, acidity, nutrient composition, and microclimatic conditions, significantly influence plant growth, biomass distribution, and the composition of plant communities [61,62,63]. Variations in soil moisture, temperature, and local microclimates further shape plant community structures and microbial ecosystems, demonstrating the complex interdependence between soil and vegetation [62,63,64].

Plants, in turn, contribute to the shaping of soil properties. For instance, plant species can leave lasting “legacies” in the soil, influencing the development of subsequent plant communities. These plant–soil feedback interactions are vital for understanding ecosystem dynamics and nutrient cycling [61,62,65]. Additionally, invasive plant species can alter soil organic matter dynamics and microbial composition, further emphasizing the bidirectional relationship between plants and soils [61,62,66].

From a wide-ranging perspective, soil health profoundly impacts plant productivity and resilience through three interrelated dimensions: physical, chemical, and biological. Physically, soil structure affects water retention, aeration, and root penetration. Well-textured and porous soils ensure adequate water and oxygen availability, which is crucial for root health and overall plant vigor. Chemically, nutrient availability, soil pH, and the balance of essential minerals like nitrogen, phosphorus, and potassium determine the soil’s capacity to support crop growth. Nutrient deficiencies or imbalances can weaken plants, making them more susceptible to stress [67].

Biologically, soil health hinges on microbial activity, organic matter decomposition, and symbiotic relationships, such as those between nitrogen-fixing bacteria and legumes or mycorrhizal fungi and plant roots. These interactions enhance nutrient cycling, improve soil structure, and strengthen plant defenses against pathogens. Soils rich in microbial diversity create a favorable environment for plant growth by improving nutrient uptake and suppressing harmful microbes [68].

Given the interconnection of soil and plant systems, fostering soil health is fundamental for sustainable agriculture. Practices like crop rotation, organic amendments, and precision farming can restore and maintain soil quality, leading to enhanced crop performance and resilience to environmental stressors [53,69]. ROA, which treats soil as a dynamic living system, offers a holistic approach to improving soil and plant health. By integrating these practices, farmers can achieve long-term productivity while minimizing environmental impacts, paving the way for more resilient agricultural systems.

### 3.2. Soil Health and Food Quality

Food quality is shaped by a complex interplay of factors, including the chemical composition of food, such as the concentration of nutrients and phytochemicals, often referred to as secondary metabolites or bioactives, and the specific forms in which these compounds are present, such as their glycosylation status, which influences their bioavailability and health benefits. Equally important is food safety, defined by the absence of pathogens, mycotoxins, chemical contaminants, and toxic levels of minerals or phytochemicals [70]. Organoleptic qualities, including taste, flavor, aroma, visual appearance, texture, and storage stability, also play a significant role in determining food quality. Additionally, practical attributes, such as the food’s suitability for handling and distribution along the supply chain, contribute to its overall value [67,70].

Soil health plays a key role in determining the nutritional quality of food. A healthy soil ecosystem, characterized by high biodiversity and robust microbial activity, enhances nutrient cycling and improves the bioavailability of essential minerals and phytochemicals in crops [71,72,73]. Conversely, conventional agricultural practices, such as intensive tillage, synthetic nitrogen fertilization, and pesticide use, disrupt the symbiosis between plants and soil microbiota, leading to a decline in the nutritional density of crops [8,54,74]. In contrast, ROA’s methods promote soil biodiversity, resulting in higher levels of vitamins, minerals, and phytochemicals in the food produced, although the underlying mechanisms remain an area for further exploration [8].

The influence of soil types on plant biochemical composition further highlights the importance of soil health in food quality. These are just a few examples that illustrate this relationship. Research on wild garlic (*Allium ursinum*) demonstrated that leaves grown in Chernozem soil exhibited the highest levels of phytochemicals and antioxidant potential [75]. Similarly, lemongrass cultivars showed significant variations in biochemical properties, yield, and quality based on soil texture, underlining the critical role of soil characteristics in plant development [76]. Moreover, studies on *Lepidozia borneensis* extract revealed that soil type influences antioxidant and anti-proliferative activities, while marine plants like red seaweed (*Kappaphycus alvarezii*) and seagrass (*Cymodocea serrulata*) displayed distinct phytochemical profiles and antioxidant properties depending on soil composition [77,78]. These findings emphasize that soil characteristics are not only critical for plant growth but also for determining the bioactive properties and nutritional quality of agricultural produce.

Cover crops and organic amendments are essential tools for improving soil fertility and, subsequently, food quality. By preventing soil erosion, enhancing moisture retention, and fostering microbial biodiversity, these practices improve nutrient availability in the soil, which directly translates to higher concentrations of bioactive compounds in crops [79,80].

Soil microorganisms, particularly arbuscular mycorrhizal fungi (AMF) and plant growth-promoting rhizobacteria (PGPR), play a crucial role in nutrient cycling, nitrogen fixation, and phosphate solubilization, further improving the nutritional composition of food [81]. A promising approach to further enhance soil biodiversity and nutrient efficiency is the application of biofertilizers, which provide a sustainable alternative to chemical fertilizers. Phosphate-solubilizing microorganisms mobilize bound phosphates, making them more accessible to plants, while nitrogen-fixing bacteria convert atmospheric nitrogen into plant-usable forms, significantly improving soil fertility [82,83]. Additionally, algal biofertilizers supply essential nutrients, enhance soil structure, and promote microbial activity and water retention [84]. Similarly, fungal biofertilizers, such as mycorrhizal fungi, establish symbiotic relationships with plant roots, improving nutrient absorption and overall plant health [85,86].

Integrating biofertilizers and microbial inoculants into agricultural systems has been shown to increase the concentration of bioactive compounds in crops, thereby enhancing their nutritional profile and health benefits [87]. For instance, studies have shown that plants grown in soils enriched with mycorrhizal fungi exhibit increased levels of essential minerals and antioxidants [88,89,90]. Likewise, PGPR-inoculated crops often show improved vitamin content and greater stress resilience, resulting in more nutrient-dense and health-promoting foods [91,92].

Adopting strategies such as biofertilizer application, promoting mycorrhizal associations, and enriching rhizosphere microbiome diversity is a key step toward sustainable agriculture. These approaches not only support soil regeneration and crop productivity but also enhance food quality, increase nutrient density, and reduce dependence on synthetic inputs, fostering more resilient and environmentally friendly farming systems [85,86]. Moreover, sustainable soil management practices, such as integrated nutrient management and conservation tillage, improve soil structure and nutrient retention while reducing reliance on chemical inputs. These methods enhance the concentration of essential nutrients and bioactive compounds in crops while mitigating environmental degradation and ensuring food safety [93].

In summary, the health of the soil is intricately linked to the quality of the food it produces. By prioritizing soil biodiversity and functionality, agricultural systems can improve both the nutritional value and safety of food, ultimately benefiting consumer health and ensuring long-term sustainability.

### 3.3. Effects on Food Quality

ROA aims to eliminate genetically modified organisms (GMOs), antibiotics, pesticides, and mineral fertilizers in order to enhance soil biological activity, balance nutrients, improve regenerative properties, and increase crop yields [24,94]. This approach leads to environmentally friendly products free of chemical elements not naturally present in food. RA focuses on restoring soil health, biodiversity, and ecosystem services, aiming to increase soil organic carbon levels and enhance water cycles, which can lead to improved soil health, increased resilience to climate change, cost savings, and sustainably produced food that meets consumer demand [24,53,94].

By restoring soil function through ROA practices, agricultural systems can overcome nutritional limitations, improve physical properties, increase soil organic matter, and foster beneficial microbial communities [53,95]. These practices also aim to achieve sustainable intensification, resilience, and long-term food security by transforming the foundation of agricultural systems [94,96]. By optimizing resource use, precision farming, and promoting sustainable practices, ROA approaches contribute to economic viability, environmental sustainability, and food security [24,53,94]. This transformative method not only supports farmers’ livelihoods but also aligns with the broader goals of environmental protection and sustainable development, creating more resilient and sustainable food systems globally [94,96,97].

In particular, agricultural management practices, such as organic farming and crop rotation, play a crucial role in determining the polyphenol content of crops, which directly influences their nutritional quality [98,99]. Adequate soil management techniques, including the use of organic amendments and micronutrient fertilization, can contribute to increased polyphenol synthesis in plants [100]. Furthermore, the selection of plant varieties with high inherent polyphenol content and the optimization of growing conditions through proper irrigation and pest control strategies can further enhance polyphenol levels in agricultural produce [101,102]. By focusing on agricultural management strategies that prioritize polyphenol accumulation in plants, farmers can potentially improve the nutritional quality of their crops and meet consumer demand for healthier food options.

#### 3.3.1. Influence on Macronutrient Content

Unlike conventional systems, which often prioritize yield maximization through synthetic inputs, ROA focuses on soil health and nutrient cycling, leading to improved macronutrient density in edible plant tissues. The enrichment of soil organic matter, coupled with increased microbial activity and enhanced nutrient availability, plays a crucial role in optimizing plant metabolism and macronutrient synthesis [103].

One of the advantages of regenerative farming is its ability to improve nitrogen mineralization, a process through which organic matter decomposition releases nitrogen in plant-available forms [23]. This mechanism is particularly relevant for protein synthesis, as nitrogen is a fundamental component of amino acids, the building blocks of proteins essential for human nutrition [104]. The slow and sustained release of nitrogen from compost and organic amendments supports balanced protein accumulation in crops, contrasting with the rapid nutrient depletion often observed in conventionally fertilized soils [94,105,106].

Carbohydrate composition is also influenced by soil health and nutrient availability. Research suggests that ROA systems promote greater starch and soluble sugar accumulation in root and tuber crops, such as potatoes and sweet potatoes, likely due to improved soil structure and water retention capacity [4,94,107]. Similarly, enhanced microbial diversity and nutrient cycling in regenerative systems facilitate higher fiber content in whole grains, fruits, and vegetables, contributing to improved gut health and metabolic regulation in human diets [106,108]. The presence of beneficial soil microbiota stimulates the synthesis of bioactive polysaccharides, which strengthen plant defense mechanisms and improve crop quality by enhancing carbohydrate composition and fiber content. These compounds contribute to digestive health and metabolic balance in human diets [109]. For instance, exopolysaccharides (EPSs) secreted by various microorganisms contribute to soil structure stabilization, nutrient entrapment, and microbial community resilience, allowing plants to better tolerate abiotic stress. These EPSs not only promote plant growth but also have applications beyond agriculture, including potential biomedical uses such as immune modulation and antitumor activity [110]. The ability of EPS-producing microbes to compartmentalize the soil microbial community further enhances their role in plant stress tolerance, making them valuable in the context of sustainable agriculture [109,110,111,112].

Beyond individual macronutrients, ROA fosters a more balanced macronutrient profile in crops, which is critical for both food quality and human health. Comparative studies on conservation agriculture and organic farming indicate that these systems produce grains with higher protein content and better essential amino acid balance, as well as fruits and vegetables with greater dietary fiber and carbohydrate complexity [54,113]. These improvements are attributed to soil organic matter retention, microbial activity, and nutrient cycling efficiency, which collectively optimize macronutrient synthesis and storage in plant tissues.

Although more research is needed to further quantify how ROA systems influence macronutrient density, key gaps remain in understanding the variability across different crop species, climatic conditions, and soil types. Comparative studies evaluating long-term effects on staple crops versus specialty crops, as well as the interactions between regenerative practices and external environmental factors, will be essential to fully assess the scalability and consistency of these benefits.

#### 3.3.2. Influence on Micronutrient Content

Micronutrient deficiencies (MNDs), resulting from inadequate intake of essential nutrients such as calcium (Ca), iron (Fe), magnesium (Mg), iodine (I), selenium (Se), potassium (K), and zinc (Zn), affect over two billion people worldwide and pose a significant challenge to achieving the United Nations’ zero hunger goal by 2030 [114,115,116]. Since plants obtain micronutrients primarily from the soil, agricultural practices that enhance soil health play a crucial role in determining the nutrient density of crops [117]. ROA systems, which prioritize biodiversity, soil structure, and nutrient cycling, have been shown to improve the bioavailability of essential micronutrients in food crops. Unlike conventional agriculture, which often relies on synthetic fertilizers and intensive tillage, ROA promotes soil organic matter accumulation, microbial activity, and efficient nutrient cycling, all of which contribute to improved micronutrient uptake by plants, which can help mitigate MNDs. Notably, Zn, a vital cofactor for numerous enzymes involved in immune function and DNA synthesis, is frequently found in greater amounts in crops grown in soils enriched with compost and organic fertilizers [118,119]. Similarly, regenerative agricultural practices that enhance soil biodiversity improve the bioavailability of key micronutrients such as Mg, K, and Fe, which play fundamental roles in enzymatic activity, energy metabolism, and oxygen transport. The application of compost, cover cropping, and minimal soil disturbance increases the availability of micronutrients by enhancing soil structure and microbial interactions [94,105,106]. Microbial communities play a fundamental role in mineral solubilization and nutrient exchange, releasing essential elements from organic and mineral sources and making them more accessible to plants [107,120].

One of the key mechanisms through which ROA improves nutrient density is by increasing the cation exchange capacity (CEC), a measure of the soil’s ability to retain and supply essential cations such as Ca^2^⁺, Mg^2^⁺, K⁺, and Fe^3^⁺. Higher CEC values enhance soil fertility by preventing nutrient leaching and ensuring a steady supply of essential cations like Ca^2^⁺, Mg^2^⁺, and K⁺ to plant roots. This improved nutrient retention translates to higher micronutrient concentrations in edible crops [121]. Additionally, diverse plant-microbe interactions in biologically active soils facilitate the synthesis of natural chelators, such as organic acids and siderophores, which increase the solubility and absorption of essential minerals [94,107].

Empirical studies demonstrate that ROA management practices enhance the micronutrient content of staple crops such as rice, wheat, and tomatoes. Crops grown in organically managed soils have been found to contain higher concentrations of Zn, Fe, and Mg, largely due to the improved soil structure and microbial diversity that facilitate nutrient availability [106,108]. In rice, regenerative practices such as cover cropping and organic amendments have been associated with increased Zn accumulation in grains [115,122]. Similarly, tomatoes cultivated under organic fertilization and deficit irrigation conditions exhibit elevated vitamin C levels and improved mineral content [123,124].

Outside individual nutrient enhancements, regenerative systems also contribute to holistic improvements in food nutrient density. Studies comparing conservation agriculture with conventional methods indicate that regenerative farming leads to higher concentrations of phytochemicals, vitamins, and minerals in a variety of crops, though specific outcomes depend on environmental conditions and crop species [8,54,125]. Organic crops have been found to contain significantly higher levels of vitamin C, Fe, Mg, and phosphorus (P) while also showing reduced nitrate concentrations compared to conventionally grown counterparts [113]. Some studies also highlight the superior polyphenol content in organically grown fruits and vegetables, which further enhances their antioxidant potential [126,127,128,129].

Despite these promising findings, further research is needed to quantify how regenerative practices influence crop nutrient density under different soil compositions, climatic conditions, and cropping systems. Understanding the long-term effects of ROA on micronutrient bioavailability, particularly in nutrient-poor or degraded soils, is crucial for optimizing sustainable agricultural practices. The interplay between soil microbiome diversity, organic matter decomposition, and mineral bioavailability remains an area of active study, with important implications for sustainable food production and global nutrition security. Nonetheless, the growing body of evidence suggests that ROA systems offer a viable pathway to enhancing food nutrient density, reducing dependence on synthetic inputs, and improving long-term soil health. By prioritizing soil regeneration, these systems not only support higher micronutrient availability in crops but also contribute to broader environmental and public health benefits.

#### 3.3.3. Influence on Bioactive Molecule Synthesis

Soil quality is a fundamental determinant in the accumulation of bioactive compounds in crops. The physical, chemical, and microbiological properties of soil directly influence nutrient availability, water retention, and microbial interactions, all of which play a key role in the biosynthesis of polyphenols, flavonoids, and carotenoids [105,107,130]. ROA systems, which prioritize soil health through increased organic matter content and biodiversity, create optimal conditions for the production of these secondary metabolites [105,107].

Several studies have demonstrated that organically managed soils promote the synthesis of phenolic compounds and antioxidant molecules across various crops, including fruits, vegetables, and grains [106,107,130]. The presence of diverse microbial communities enhances nutrient availability and activates the plant metabolic pathways responsible for bioactive compound synthesis. The overall result is a higher concentration of these beneficial compounds, which contribute not only to plant adaptation and resistance but also to improved nutritional quality for consumers [94,107]. Numerous studies have shown that higher levels of these compounds contribute to antioxidant, anti-inflammatory, and cardioprotective effects, which are essential for the prevention of chronic diseases [130,131,132].

Research has shown that organically cultivated crops contain higher levels of bioactive molecules compared to conventionally grown ones [94,108,133]. This trend has been observed across a wide range of plant species, including leafy greens, cruciferous vegetables, tomatoes, berries, cereals, and legumes [94,105,107]. For example, organically grown cruciferous vegetables tend to have increased glucosinolates and flavonoids, which contribute to their antioxidant properties and potential cancer-preventive effects [107,108]. Similarly, organic tomatoes and berries have been found to exhibit higher concentrations of lycopene, anthocyanins, and polyphenols, compounds known for their role in reducing oxidative stress and promoting cardiovascular health [94,106,107]. A study conducted in Guanajuato, Mexico, further supports these findings, demonstrating that organically managed soils contained higher levels of moisture, organic matter, phosphorus, and nitrogen [108]. These enriched conditions facilitated the accumulation of phenolic compounds such as catechin, gallic acid, and rutin in various crops, reinforcing the idea that organic farming enhances the synthesis of bioactive molecules through improved soil conditions [94,108].

Beyond organic farming, regenerative practices such as minimal tillage, cover cropping, composting, and biochar application have been shown to further enrich soil fertility and microbial ecosystems, leading to greater bioactive compound production in plants [94,107,120]. These methods stimulate root–microbe interactions, facilitating nutrient uptake and secondary metabolite synthesis [105,106]. Additionally, they help to reduce oxidative stress in plants, thereby promoting the accumulation of antioxidants like flavonoids and carotenoids [94,107].

The integration of regenerative techniques also contributes to a more resilient agricultural system [23]. The use of biochar-based ecological farming, for instance, has been found to improve soil carbon content, microbial richness, and nutrient availability, leading to increased polyphenol levels in a variety of crops [107,120]. Similarly, conservation agriculture methods that reduce chemical inputs while maintaining high organic matter levels have been associated with higher nutrient density in fruits, vegetables, and grains [23,94,106]. These approaches create a self-sustaining environment that not only enhances plant metabolism but also promotes ecological balance and long-term agricultural sustainability [94,105].

The evidence indicates that ROA significantly increases the bioactive molecule content in crops, resulting in food products with enhanced nutritional value and potential health benefits. By promoting soil biodiversity, improving nutrient availability, and minimizing dependence on synthetic inputs, these agricultural practices foster an environment that naturally stimulates the synthesis of polyphenols, flavonoids, and carotenoids.

To provide a clear and structured overview of the main effects of ROA on the nutritional quality of crops, Table 1 summarizes key findings from the literature. This synthesis highlights the differences between agricultural practices in terms of nutrient and bioactive compound content, offering valuable insights into their potential health benefits.

## 4. Food-Crop Quality and Human Health

As detailed in Section 3.3.3, ROA enhances the synthesis of antioxidant compounds such as polyphenols and carotenoids in crops due to improved soil conditions and microbial interactions. In this section, we focus on the functional implications of these compounds for human health, particularly their role in oxidative stress reduction and chronic disease prevention.

### 4.1. Impact on Human Health

The impact of ROA extends beyond environmental sustainability, offering significant benefits for human health through improved nutritional profiles of food. These agricultural practices prioritize soil health, biodiversity, and reduced chemical inputs, which directly influence the macronutrient and micronutrient content of crops, enhance bioactive compounds, and boost antioxidant properties [54,70].

Numerous studies show that organically grown fruit, vegetables, and cereals contain higher amounts of essential micronutrients, antioxidants, and polyphenols [10,67,99,113,115,137,138]. These compounds help reduce oxidative stress, inflammation, and the risk of chronic diseases [132,133,138]. Additionally, ROA practices enhance nutrient uptake in plants, making them more resistant to disease [139]. Reducing reliance on synthetic pesticides and herbicides further mitigates the obesogenic risk associated with early life endocrine disruption exposure, neurotoxicity in neonatal exposed organisms and their progenies, and carcinogenicity due to chronic chemical exposure, presenting significant public health benefits [140,141]. Moreover, pasture-based livestock systems, integral to RA, yield animal products with a more favorable lipid profile, including higher levels of omega-3 fatty acids and conjugated linoleic acid, known for their cardioprotective and anti-inflammatory properties [142,143,144].

While substantial research supports the benefits of ROA for human health, gaps remain in fully understanding the long-term implications of these practices on food composition and disease prevention. More longitudinal studies and clinical trials are needed to establish definitive links between ROA diets and health outcomes. However, the growing body of evidence reinforcing the connection between soil health, nutrient density, and human well-being highlights the transition to organic and regenerative systems as an imperative strategy for promoting long-term health while safeguarding environmental integrity.

### 4.2. Antioxidant-Related Benefits

As we have already pointed out, foods grown using ROA methods often exhibit superior antioxidant properties. Antioxidants are crucial for neutralizing reactive oxygen species (ROS), which are associated with aging and numerous diseases, including cancer [145,146]. Emerging evidence suggests that ROS play a crucial role as signaling molecules throughout the entire cell death pathway [147]. Excessive ROS production can damage organelle structures and biomolecules, triggering an inflammatory response, a well-established underlying mechanism in the development of diabetes and cancer [147,148]. Crops from organically managed and regenerative systems consistently show higher levels of antioxidants such as vitamin C, tocopherols, and glutathione [54,120]. These outcomes are linked to enhanced soil microbiota activity and increased availability of nutrients like selenium, a key component of glutathione peroxidase (GPx), an enzyme that protects cells from oxidative damage [149,150]. Additionally, avoiding synthetic pesticides and fertilizers minimizes chemical stress on plants and humans, prompting them to enhance natural defense mechanisms [151]. This shift leads to greater investment in antioxidant biosynthesis, particularly in secondary metabolites such as flavonoids and phenolic compounds [74,151,152].

Building on these findings, specific bioactive compounds found in organically grown foods, such as lycopene in tomatoes, play a crucial role in modulating oxidative stress and inflammation, further highlighting the health benefits of antioxidant-rich diets [3]. Tomatoes are rich in lycopene, a red carotenoid pigment with potent antioxidant properties [99,153]. Lycopene scavenges singlet oxygen and peroxyl radicals, reducing oxidative stress. It also inhibits nuclear factor-kB (NF-κB) signaling, a pro-inflammatory pathway, thereby exerting anti-inflammatory effects. Furthermore, lycopene enhances the nuclear factor erythroid 2-related factor (Nrf2) pathway, promoting the expression of antioxidant response element (ARE)-regulated genes such as heme oxygenase-1 (HO-1) and superoxide dismutase (SOD), which help detoxify ROS and maintain cellular redox balance [147,153]. Moreover, lycopene supports cardiovascular health, reduces cancer risk, combats oxidative stress, lowers blood pressure, prevents LDL oxidation, and inhibits tumor growth [153]. Its antioxidant properties also benefit the skeletal system and neurodegenerative diseases like Alzheimer’s and Parkinson’s [153,154].

Similarly, polyphenols found in organic fruits, such as quercetin in apples and flavonoids in berries, contribute to antioxidant defense by modulating key enzymes in ROS metabolism [155]. Quercetin inhibits xanthine oxidase, an enzyme involved in ROS production, and upregulates catalase (CAT) and GPx, two critical enzymes that neutralize hydrogen peroxide (H_2_O_2_) [155]. In addition, quercetin interacts with SIRT1, a sirtuin involved in longevity and metabolic regulation, contributing to antiaging effects by enhancing mitochondrial function and cellular resilience against oxidative stress [144,156].

Although a wide range of crops—including cereals, legumes, cotton, and vegetables—may benefit from regenerative organic agriculture [69,157,158,159,160,161], we deliberately focused on well-documented examples, such as grapes and turmeric, where the literature clearly illustrates the connections between soil health, phytochemical composition, and specific antioxidant or anti-inflammatory pathways [108,137,162].

On the anti-inflammatory side, curcumin, a bioactive compound in turmeric, modulates inflammatory pathways by inhibiting cyclo-oxygenase-2 (COX-2) and nitric oxide synthase (iNOS), enzymes responsible for prostaglandin (PG) and nitric oxide (NO) synthesis, respectively [163,164]. This leads to reduced production of pro-inflammatory cytokines such as TNF-α and IL-1 [165]. Curcumin also suppresses the activation of NF-κB, a key regulator of inflammation that controls the expression of numerous pro-inflammatory genes [166]. By modulating NF-κB activity, curcumin reduces the transcription of inflammatory mediators, thereby alleviating chronic inflammation linked to various diseases, including arthritis, metabolic syndrome, and neurodegenerative disorders [166]. Additionally, curcumin enhances the activity of peroxisome proliferator-activated receptor-gamma (PPAR-γ), a nuclear receptor involved in lipid metabolism and inflammation resolution, further contributing to its protective effects [167].

Beyond its role in inflammation suppression, curcumin activates AMP-activated protein kinase (AMPK), promoting mitochondrial biogenesis and cellular energy balance, which further supports its antiaging properties [164]. This activation improves insulin sensitivity, enhances fatty acid oxidation, and regulates glucose homeostasis, making curcumin a promising therapeutic agent for metabolic disorders such as type 2 diabetes and obesity [154]. Furthermore, curcumin modulates the gut microbiota composition, promoting the growth of beneficial bacteria while inhibiting pathogenic strains, thereby reducing intestinal inflammation and contributing to overall health [168].

Recent agronomic research supports the claim that agricultural practices, particularly organic and regenerative methods, significantly influence curcumin content in *Curcuma longa*. Organic systems—leveraging compost, vermicompost, and biofertilizers—create favorable soil conditions that enhance secondary metabolite synthesis. For example, [169] found that vermicompost application, either alone or with phosphate-solubilizing bacteria and *Azospirillum*, significantly improved turmeric rhizome biomass and curcumin content. Similarly, [170] demonstrated that integrating biofertilizers with organic inputs like neem cake and compost enhanced both yield and curcumin concentration, achieving up to 6.32% curcumin content. In another study, [162] reported that combining 50% of the recommended doses of synthetic fertilizers with neem compost improved curcumin levels (5.13%) while maintaining soil fertility, underscoring the value of integrative approaches. While direct evidence on regenerative agriculture remains limited, sustainable strategies like reduced tillage and cover cropping are known to foster favorable soil conditions that could similarly promote curcumin synthesis through enhanced organic matter and microbial diversity [162]. Therefore, organic and regenerative farming approaches hold promise for improving not only the agronomic performance and environmental sustainability of turmeric cultivation but also its nutritional and therapeutic properties.

Emerging research also highlights curcumin’s role in epigenetic modulation, as it influences histone modifications and DNA methylation patterns, potentially impacting gene expression related to inflammation and aging [171]. These results suggest that curcumin not only provides immediate anti-inflammatory benefits but also induces long-term protective effects at the molecular level, underscoring its potential as a multi-faceted therapeutic compound for chronic diseases and longevity.

**Figure 3:**
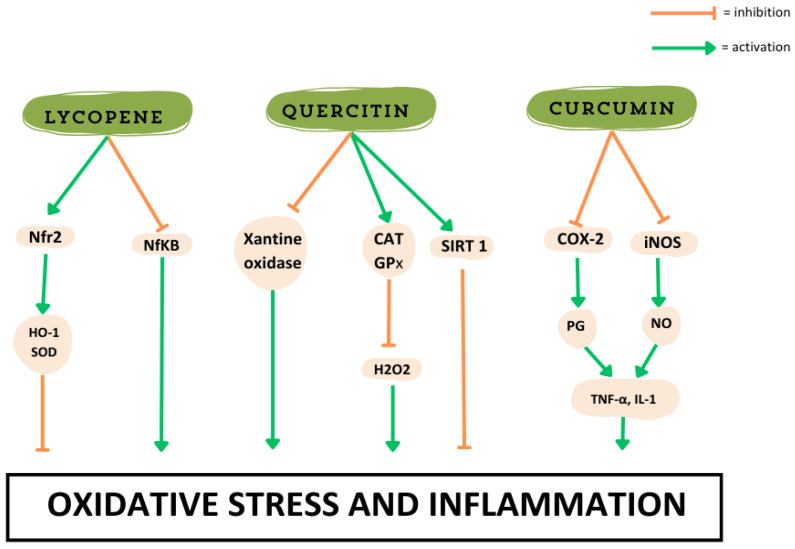
Examples of molecules enriched from ROA and pathways of activation in reducing oxidative stress and inflammation. Original figure created by the authors.

These findings suggest that even slight increases in specific bioactive compounds, influenced by soil quality and agricultural practices, can significantly enhance the biochemical profile of food. Consequently, consuming such nutrient-rich foods can not only modulate metabolic pathways but also strengthen immune defenses and promote cellular resilience against aging and chronic diseases. Moreover, since the antioxidant and anti-inflammatory properties of these compounds are closely interconnected, they work together to mitigate oxidative stress and reduce chronic inflammation, both of which are key factors in disease prevention and longevity. These results underscore the potential health benefits of ROA products, which, through their enriched bioactive and nutrient composition, help regulate oxidative stress, inflammation, and aging-related pathways (Figure 3). By enhancing soil health and promoting natural plant defenses, ROA not only improves food quality but also contributes to disease prevention and longevity.

## 5. Conclusions and Future Perspectives

ROA provides measurable health benefits by improving the macronutrient and micronutrient content of food, enhancing bioactive molecules, and boosting antioxidant properties. These advantages are deeply rooted in the improved soil health and biodiversity associated with these sustainable practices. By prioritizing these agricultural systems, we can not only protect the environment but also promote human health and reduce the burden of diet-related diseases.

There is growing scientific interest in the relationship between agricultural practices, soil health, food quality, and human well-being, with increasing evidence supporting the role of ROA in building more sustainable and nutrient-rich food systems. These approaches prioritize soil restoration, biodiversity conservation, and ecological balance, reducing reliance on synthetic inputs while enhancing the availability of essential nutrients and bioactive compounds in crops.

Soil health is at the core of this paradigm shift, as it directly influences plant metabolism, nutrient composition, and the synthesis of bioactive molecules such as polyphenols, flavonoids, and carotenoids. A well-structured soil ecosystem, enriched by microbial diversity and organic matter, enhances plant resilience, supports sustainable yields, and contributes to improved food quality with potential health benefits [74]. Scientific studies confirm that crops grown in regenerative and organic systems often exhibit higher concentrations of vitamins, minerals, and antioxidants, with implications for disease prevention and overall human health. This is of particular interest considering that consuming nutrient-dense foods has been associated with a modestly decreased risk of cardiovascular diseases, diabetes, and all-cause mortality [172]. Thus, by increasing nutrient density, ROA may offer a promising strategy to combat non-communicable diseases, which remain one of the leading global health challenges.

Beyond nutritional aspects, ROA has the potential to mitigate climate change through carbon sequestration and improved soil structure while reducing the environmental footprint of conventional farming. Sustainable practices, such as cover cropping, crop rotation, reduced tillage, and livestock integration, play a fundamental role in restoring degraded ecosystems and increasing farm resilience.

As global food systems face mounting pressures, transitioning to a more sustainable and health-focused model of agriculture is no longer an option but a necessity. By advancing scientific research, promoting innovative farming techniques, and fostering a deeper understanding of the connections between soil, food, and health, ROA can pave the way toward a more resilient, environmentally responsible, and nutritionally beneficial future.

This approach offers a promising pathway to align agricultural productivity with sustainability and public health goals. However, its widespread adoption will require supportive policy frameworks that incentivize sustainable soil management, promote biodiversity, and ensure equitable access to regenerative practices—particularly for smallholders and transitional systems. Integrating ROA principles into national and international agri-food policies could contribute to long-term food security and public health resilience.

From a research perspective, more interdisciplinary studies are needed to evaluate the specific health impacts of crops grown under ROA systems. Clinical trials, nutritional metabolomics, and long-term epidemiological studies would help clarify the mechanisms linking soil health to human physiology. Additionally, better standardization of RA/ROA definitions and metrics is crucial to foster comparability across studies and support evidence-based policymaking. Overall, ROA offers a unique opportunity to reconnect agricultural practices with the broader goals of planetary and human health.

## Figures and Tables

**Table 1:** Summary of selected studies reporting the effects of different agricultural practices on crop nutritional quality and bioactive compound content. Please check supplementary materials for extended data (Table S1).

Reference	Crop(s)	Treatment/Soil Management	Key Finding
[74]	Various crops	Organic vs. conventional	↑ Antioxidants, ↓ cadmium, ↓ pesticide residues
[134]	Cereals (e.g., wheat)	Zn-enriched fertilizers	↑ Zinc and iron contents in grains
[135]	Fruits and vegetables	Organic and no-tillage systems	↑ Polyphenols and antioxidant activity
[136]	Carrots	Thermophile-fermented compost	↑ Bioactive compounds and yield
[115]	Various crops	Regenerative practices	↑ Micronutrients and vitamin C
[100]	Fruits and vegetables	Low-nitrogen fertilization	↑ Polyphenols (especially in vegetables)
[108]	Grapes	Organic vs. conventional	↑ Phenolic compounds and antioxidant capacity
[137]	Tempranillo grapes	Different soil types (A.O.C. Rioja)	Different anthocyanin profiles based on soil composition
[113]	Fruits, vegetables, grains	Organic vs. conventional	↑ Vitamin C, Fe, Mg, P, ↓ nitrate content
[127]	Prepacked foods	Organic vs. conventional	Variable results, ↓ energy and sugars in organic versions

Note: ↑ indicates an increase; ↓ indicates a decrease.

## Supplementary Materials

The following supporting information can be downloaded at: https://www.mdpi.com/article/10.3390/antiox14050530/s1, Table S1: Comprehensive summary of studies evaluating the effects of soil type and agricultural practices on the nutritional composition and bioactive compound content of crops.

## Abbreviations

The following abbreviations are used in this manuscript:

AMF	Arbuscular mycorrhizal fungi
AMPK	AMP-activated protein kinase
ARE	Antioxidant response element
Ca	Calcium
CAT	Catalase
CEC	Cation exchange capacity
COX-2	Cyclo-oxygenase-2
EPS	Exopolysaccharide
Fe	Iron
GPx	Glutathione peroxidase
GMO	Genetically modified organisms
H_2_O_2_	Hydrogen peroxide
HO-1	Heme oxygenase-1
I	Iodine
K	Potassium
Mg	Magnesium
MND	Micronutrient deficiencies
NF-kB	Nuclear factor-Kb
NO	Nitric oxide
Nrf2	Nuclear factor erythroid 2-related factor
P	Phosphorus
PG	Prostaglandin
PGPR	Plant growth-promoting rhizobacteria
PPAR-γ	Peroxisome proliferator-activated receptor-gamma
RA	Regenerative agriculture
ROA	Regenerative organic agriculture
ROS	Reactive oxygen species
SOD	Superoxide dismutase
Se	Selenium
Zn	Zinc
