# Supplementary material for: Regenerative Organic Agriculture and Human Health: The Interconnection Between Soil, Food Quality, and Nutrition
Source: Antioxidants (Basel). 2025 Apr 29;14(5):530. doi: 10.3390/antiox14050530 (PMC12108233; doi:10.3390/antiox14050530)
Supplement: Supplementary file 1 [file antioxidants-14-00530-s001.zip › antioxidants-3598975-supplementary.pdf]

Table S1. Comprehensive summary of studies evaluating the effects of soil type and agricultural practices on the nutritional composition and bioactive compound content of crops.

This table presents a detailed overview of relevant peer-reviewed studies, including crop types, soil conditions or management systems, nutritional or phytochemical outcomes, and methodological approaches. The summarized evidence supports the role of regenerative and organic practices in enhancing nutrient density, antioxidant capacity, and food quality.

Reference	Crop type	Soil type	Nutrient/ Bioactive compound/ Soil parameters analyzed	Observed variation	Analysis methods	Main conclusions
**[73]**	Various crops	Organic vs. conventional farming	Antioxidants (polyphenolics), cadmium (Cd), pesticide residues	Higher concentrations of antioxidants, lower Cd levels, and reduced pesticide residues in organic crops	Systematic literature review and meta-analyses	Organic crops have higher antioxidant levels, lower Cd concentrations, and fewer pesticide residues compared to conventional crops
**[133]**	Various crops	Organic vs. conventional farming	Various nutrients, sensory qualities, food safety	No significant differences in most nutrient levels except for nitrates; potential differences in sensory qualities; lower pesticide residues in organic foods	Systematic literature review	No strong evidence of major nutritional differences between organic and conventional foods except for lower nitrate levels; organic foods tend to have fewer pesticide residues
**[134]**	Cereals (e.g., wheat)	Soils treated with	Zn, Fe	Increased Zn and Fe concentrati	Field experiments, soil and crop	Agronomic practices, such as Zn-

		micronutrient-enriched fertilizers		ons in grains through soil and foliar Zn fertilization; late foliar Zn application is particularly effective	nutrient analysis	enriched fertilization, are effective strategies for improving the biofortification of cereals with essential micronutrients
**[135]**	Fruits and leafy vegetables	Integrated, organic, and organic no-tillage farming systems	Bioactive compounds (e.g., polyphenols, flavonoids), antioxidant activity	Variations in bioactive compound concentrations and antioxidant activity depending on the farming system	Two-year rotation experiment, chemical analysis of bioactive compounds	Organic and organic no-tillage practices can enhance the bioactive properties of fruits and leafy vegetables compared to integrated farming systems
**[126]**	Various prepacked foods	Not applicable	Energy, macronutrients, salt	Few significant differences between organic and conventional products; organic "jams, chocolate spreads, and honey" had lower energy, carbohydrates, sugars, and higher protein; organic "pasta, rice, and other cereals" had lower energy,	Systematic survey of 569 pairs of organic and conventional products	Organic certification does not necessarily indicate superior nutritional quality; differences exist in specific product categories

				protein, and higher saturates		
**[136]**	Various vegetables (e.g., parsley, beet, celery, carrot, onion, leek, potato)	Organic vs. conventional farming systems	Macro elements (Ca, Mg, Na, N, K, P)	Organically grown vegetables generally exhibited higher concentrations of macro elements compared to conventionally grown counterparts; soil samples from organic farms also showed higher levels of these elements	Comparative analysis of vegetable and soil samples from organic and conventional farms	Organic farming practices may enhance the accumulation of essential macro elements in vegetables, potentially leading to improved nutritional quality
**[137]**	Various agricultural produce	Organic vs. conventional farming systems	Nutritional value, pesticide residues, heavy metals, mycotoxins	Organic produce tends to have higher antioxidant levels and lower pesticide residues; findings on heavy metals and mycotoxins are mixed	Systematic literature review	Organic farming can enhance certain aspects of food quality, but results vary; more research is needed to draw definitive conclusions
**[98]**	Selected tomato types	Organic vs. conventional cultivation systems	Nutritional value parameters, phenolic compounds	Organic cultivation resulted in higher levels of certain phenolic compounds	Comparative analysis of tomatoes from different cultivation systems	Organic growing systems affect tomato quality parameters, including

				; variations in nutritional value parameters were observed between cultivation systems and tomato types		nutritional value and phenolic compound content; tomato type also significantly influences these parameters
**[99]**	Fruits and vegetables	Various soil types under different fertilization and management practices	Polyphenols (flavonoids, anthocyanins)	Generally, lower nitrogen fertilization is associated with higher polyphenol content; agricultural practices have a stronger influence on polyphenol levels in vegetables than in fruits	Literature review	Nitrogen fertilization and agricultural practices significantly affect polyphenol content in plants; reduced nitrogen input tends to increase polyphenol levels, with a more pronounced effect in vegetables
**[6]**	Cotton-based crop rotation	Organic vs conventional farming in Vertisol (India)	Soil organic carbon, nitrogen content, microbial abundance and activity	Increased soil organic carbon and nitrogen content; enhanced microbial abundance and activity; distinct microbial community composition in organic farming systems	Field experiments comparing organic and conventional farming systems	Organic farming enhances soil quality and fosters unique microbial communities in cotton-based rotations on Indian Vertisols

**[107]**	Grapes (Vitis vinifera L.) varieties Syrah and Tempranillo	Organic vs. conventional vineyards in Guanajuato, Mexico	Phenolic compounds (myricetin, quercetin, resveratrol, ellagic acid), antioxidant capacity	Organic vineyards had higher soil moisture, clay content, organic matter, phosphorus, nitrogen, and oxides; grapes from organic vineyards exhibited higher levels of phenolic compounds and antioxidant capacity	Soil analysis, HPLC for phenolic profiling, antioxidant assays, principal component analysis	Organic agriculture enhances soil quality and increases the concentration of bioactive compounds in grapes, improving their suitability for winemaking
**[114]**	Various crops (e.g., rice, wheat, tomato)	Various soils under regenerative practices	Micronutrients (e.g., Zn, Fe), vitamin C	Increased concentrations of micronutrients and vitamin C in edible portions under specific regenerative practices	Scoping review of literature from 2000–2021	RA practices, such as increased organic inputs and deficit irrigation, can enhance micronutrient concentrations in crops, though effects are context-specific and vary by crop type
**[9,114]**	Various fruits	Organic vs. conventional farming systems	Nutritional composition, bioactive compounds, postharvest quality parameters	Organic fruits often exhibit higher levels of certain bioactive compounds	Systematic literature review	Organic farming practices can enhance the nutritional quality and postharvest attributes of

				and antioxidants; variations in postharvest quality attributes between organic and conventional fruits		fruits, though results vary depending on fruit type and specific agricultural practices
**[75]**	Lemongrass (Cymbopogon flexuosus)	Different soil types (alkaline and non-alkaline soils)	Essential oil content, citral, geraniol, biochemical constituents	Variations in essential oil yield and composition; alkaline soils influenced biochemical profiles	Field experiments analyzing yield, oil composition, and biochemical parameters	Soil type significantly affects the yield and quality of lemongrass, with alkaline soils impacting essential oil composition and biochemical constituents
**[138]**	Carrots	Soil enriched with thermophile-fermented compost	Antioxidant activity, bioactive compounds	Increased productivity, enhanced antioxidant activity, and higher bioactive compound content	Chemical and biochemical analysis of carrot samples	The application of thermophile-fermented compost significantly improves crop yield and nutritional quality
**[7]**	Various food crops	Organic vs. conventional farming systems	Soil microbial biomass, activity, diversity; nutrient density in crops	Organically farmed soils exhibited greater microbial biomass, activity, and diversity; potential implication	Systematic literature review	Organic farming practices enhance soil health parameters, which may contribute to improved nutrient density in crops

				s for nutrient density in crops		
**[53]**	Various food crops	RA vs. conventional farming	Mg, Ca, P, Zn, vitamins (B1, B12, C, E, K), phytochemicals	Increased nutrient and phytochemical levels in regenerative farming crops	Comparative analysis of paired farms across the U.S. practicing regenerative or conventional farming for 5-10 years	RA enhances mineral and vitamin content in food crops, supporting improved nutritional quality and indicating potential benefits for human health
**[67]**	Solaris grapes (Vitis vinifera L.)	Organic vs. conventional farming systems	Polyphenols, antioxidant activity, pesticide residues, microbiological properties	Organic grape juice exhibited higher polyphenol content and antioxidant activity; differences in microbial diversity; presence of sulfur and copper residues in organic juice, synthetic pesticide residues in conventional juice	Chemical analysis, microbiological assays, residue testing	Organic farming enhances certain health-promoting compounds in grape juice but may result in residues from organic treatments; conventional farming associated with synthetic pesticide residues
**[139]**	Tempranillo grapes (Vitis vinifera L.)	Three different soil types (A.O.C. Rioja vineyards)	Anthocyanin composition	Significant differences in anthocyanin profiles depending on soil type	HPLC	Soil type influences the anthocyanin composition in grapes, affecting wine quality
**[66]**	Various crops	Organic vs. convention	Soil health indicators,	Organic farming		There are potential

		al farming systems	crop nutritional quality	practices enhance soil health parameters, which are linked to improved crop nutritional quality; specific outcomes depend on management practices and environmental conditions	Literature review and synthesis	links between organic farming, improved soil health, and enhanced food quality; further research is needed to elucidate these relationships
**[4]**	Various crops	Global soil types	Soil nutrients (e.g., nitrogen, organic matter)	Nitrogen- and organic-rich soils supported the highest crop yields; nutrient use efficiency was higher in regions with lower crop productivity and lower fertilizer inputs	Review of global patterns in soil characteristics and agricultural production	Soils rich in nitrogen and organic matter are crucial for high crop yields; improving nutrient use efficiency is essential, especially in regions with lower productivity
**[20]**	Various crops	Various soil types under regenerative practices	Soil health indicators, crop yield and quality parameters	Regenerative practices improved soil health metrics and influenced crop yield and quality; specific outcomes varied based on practices	Scoping review of literature on regenerative agricultural practices	Regenerative agriculture enhances soil health and can improve crop yield and quality, though effects are context-dependent; further

				and contexts		research is needed to identify optimal practices
**[112]**	Fruits, vegetables, and grains	Organic vs. conventional farming systems	Vitamin C, iron, magnesium, phosphorus, nitrates	Organic crops contained significantly more vitamin C, Fe, Mg, and P, and significantly less nitrates compared to conventional crops	Systematic literature review	There are genuine differences in the nutrient content between organic and conventional crops, with organic produce generally having higher levels of certain nutrients and lower nitrate content
